# Thromboxane-induced contractile response of mesenteric arterioles is diminished in the older rats and the older hypertensive rats

**DOI:** 10.3389/fphar.2022.1019511

**Published:** 2022-10-12

**Authors:** Min Zhang, Chunshu Li, Chunxia He, Yiqin Cui, Yuan Li, Ying Ma, Jun Cheng, Jing Wen, Pengyun Li, Yan Yang

**Affiliations:** Key Laboratory of Medical Electrophysiology of Ministry of Education and Medical Electrophysiological Key Lab of Sichuan Province, Collaborative Innovation Center for Prevention and Treatment of Cardiovascular Disease, Institute of Cardiovascular Research, Southwest Medical University, Luzhou, Sichuan, China

**Keywords:** aging-related vascular remodeling, mesenteric artery, U-46619, vasoconstriction, TXA2-TP prostaglandin signaling pathway

## Abstract

Nearly all physiological processes are controlled at some level by G-protein-coupled receptor (GPCR) signaling activity. The thromboxane A2 (TXA2) receptor (TP) is a member of the GPCR family. The ultimate effect of TP receptor activation depends on the availability of specific G proteins, which in turn depend on the cell type, tissue, and disease state. However, the roles of the TXA2-TP signaling pathway executed under disease states are poorly defined. In this study, 16-week-spontaneously hypertensive rats (SHR), the 18-month-SHR (OldSHR), and the age-matched Wistar-Kyoto (WKY) rats were used to study the vasoconstriction of mesenteric resistance artery induced by TP-specific agonist, U-46619. Vasoconstriction induced by U-46619 was significantly attenuated in OldWKY and OldSHR rats, and mesenteric arteries with impaired response to U-46619 responded strongly to the adrenergic receptor agonist, phenylephrine. Similar vascular responses to U-46619 were obtained in endothelium-denuded mesenteric arteries. Accordingly, the expression of TP membrane proteins in mesenteric vessels was decreased, and the endogenous TP competitor, 8, 9-EET, in serum was increased, which was partly responsible for the decreased vascular reactivity of U-46619. Decreased TP membrane expression was associated with TP endocytosis, which involved actin cytoskeletal remodeling, including increased ratio of F-actin/G-actin in OldWKY and OldSHR rats. Hence, we studied the effects of TXA2 and its receptors on blood vessels and found that the TXA2-TP prostaglandin signaling pathway was impaired in older adults, which would facilitate the creation of “precision therapeutics” that possess selective efficacy in diseases.

## Introduction

Prostaglandin signaling controls a wide range of biological processes, including blood pressure homeostasis, inflammation, pain, and cell survival. Thromboxane A2 (TXA2), a member of the prostaglandin family, is an arachidonic acid metabolite that is biosynthesized in cells by phospholipase A2, cyclooxygenase (COX-1 or COX-2), and TXA2 synthase (TXAS). TXA2 is a lipid mediator with potent vasoconstrictive, mitogenetic, and platelet aggregative properties (Hamberg et al., 1975; Hanasaki et al., 1990). The effects of TXA2 are mediated by specific TP and subjected to regulatory control. TP is a member of the GPCR superfamily. TP is widely expressed in various tissues and cells, including platelets, vascular systems (smooth muscle cells and endothelial cells), lung, kidney, heart, thymus, spleen, and other systems. In smooth muscle cells, activation of G protein–coupled TP induces vascular smooth muscle contraction through the initial activation of phospholipase-C (PLC), with production of 1,4,5-inositol phosphate, Ca^2+^ release from intracellular calcium stores, and phosphorylation of myosin light chains (Nakahata, 2008).

The GPCRs are known to undergo receptor desensitization following agonist stimulation (Hausdorff et al., 1990). The process involves uncoupling of G-proteins from a receptor, sequestration of receptor from cell surface, and downregulation of total receptor number (Hertel et al., 1983). Receptor phosphorylation induced by agonist stimulation is understood to contribute to each of these processes (Hausdorff et al., 1990). TP has been reported to undergo homologous desensitization after stimulation by agonists—such as U-46619 and I-BOP (Liel et al., 1988; Murray and FitzGerald, 1989; Okwu et al., 1992)—which is an agonist-dependent adaptive process in biological systems that modulates responsiveness of the cell to repeatedly stimulate over time.

TXA2 promotes various physiological responses and pathophysiological implications. Plasma TXA2 levels correlated with the prevalence of cardiovascular diseases, including hypertension (Francois et al., 2004) and atherosclerosis (Mehta et al., 1988). Furthermore, dysregulation of TP can result in agonist hypersensitivity and hyper-responsiveness, contributing to exaggerated vasoconstriction in the hypoxic pulmonary artery in neonatal persistent pulmonary hypertension (Hinton et al., 2007; Santhosh et al., 2014). Hinton et al. (2007) found that hypoxia caused sensitization of TP in neonatal pulmonary artery myocytes, but only under controlled conditions. Neonatal pulmonary artery myocytes exposed to a moderate level of hypoxia had hypersensitive and hyperresponsive peak [Ca^2+^]_i_ responses to the TXA2 agonist, U-46619, despite a reduction in cell surface TP expression. Feng et al. (2011) found that cardioplegia/reperfusion decreased the contractile response of human coronary arterioles to TXA2 soon after cardiac operations. They suggested that thromboxane-induced contractile response of human coronary arterioles is diminished after cardioplegic arrest, and the contractile response to U-46619 was due to activation of TP and PLC. Tang and Vanhoutte (2008) reported that the gene expression of prostanoid synthases in endothelial cells and prostanoid receptors changed in vascular smooth muscle cells in aging and hypertension. They concluded that whether the gene modifications caused by aging and hypertension directly contribute to endothelium-dependent contractions or vascular aging and the vascular complications of the hypertensive process remains uncertain.

Aging-related diseases are thought to kill nearly 100,000 individuals worldwide daily (Gladyshev and Gladyshev, 2016; Bakula et al., 2018). While the aging process itself is generally not considered to be a disease (Novoselov, 2017), it is a pathophysiological state that facilitates or even induces the occurrence of diseases, such as hypertension, in several older adults (Rattan, 2014). Although aging has been reported to affect vascular reactivity by altering the sensitivity and maximum effect of stimulating contractile agonists and endogenous vasodilators (Hongo et al., 1988; Novella et al., 2013; Cupitra et al., 2020), age-related changes in cardiovascular physiology and pathophysiology of GPCR have been reported as well (Schutzer et al., 2001; Sparks et al., 2013), few studies on the role of TXA2 and its receptor (TP) in older individuals and older individuals with hypertension have been reported. Using the specific stable TP-specific agonist, U-46619, we found that mesenteric vasoconstriction response induced by U-46619 was diminished remarkably in OldWKY and OldSHR. Therefore, we explored the mechanisms to understand the relationship between the vascular action caused by TXA2 and its receptor to try to further elucidate the role and significance of thromboxane and its receptor under disease conditions.

## Materials and methods

### Animals

Twelve-week-old male SHR and their age-matched male WKY rats were bought from Beijing Vital River Laboratory Animal Technology Co. Ltd. [License No. SCXK (Jing) 2019–0011]. Animals were fed until 16 weeks (adult) and 18 months (older adults) at the Animal Care Center of Southwest Medical University [license No. SCXK (Chuan) 2018–17]. They were further divided into four groups: WKY, SHR, OldWKY, and OldSHR groups. Animal experiments were performed according to the Guide for the Care and Use of Laboratory Animals approved by the Ethics Committee of Southwest Medical University. The diurnal rhythm of light was maintained, and the temperature was controlled at 25°C. The animals had *ad libitum* access to food and water during feeding. Before the experiments, blood pressure of rats was measured by femoral artery cannulation to confirm normal blood pressure or hypertension. Data on rats are shown in Supplementary Table S1.

### Preparation of mesenteric vascular rings and isometric tension recording

The tertiary branches of endothelium-denuded mesenteric artery segments were prepared using previously described procedures (Zhou et al., 2019; Cui et al., 2022) (Online Supplementary Methods). Briefly, after the endothelium was rubbed away, the arterial ring was suspended on the experimental system. The basal tension was adjusted, and 60 mM K^+^ solution (High K^+^) was used to detect vascular reactivity 60 min later. After the vessels reached maximum contraction, 1 μM acetylcholine (Ach) was used to detect endothelial activity. Additionally, in some experiments, to ensure that the endothelial function is completely removed, NG-nitro-L-arginine methyl ester (L-NAME, endothelial nitric oxide synthase inhibitor) was added to block the endothelial function on the basis of removing the endothelium by mechanical eraser. If the vasodilation caused by Ach was <20%, the vessels were considered to have no endothelial residue, and the vessel could be used for subsequent experiments. High K^+^ was repeated to make the blood vessels contract twice, and the difference between the two contractions was calculated. If the difference was <10%, the maximum contraction caused by High K^+^ was considered to be stable. Thereafter, the bath was washed with Tyrode’s solution, and the follow-up experiment was conducted after 30 min of stability.

### RNA sequencing and functional enrichment analysis

Total RNA was isolated from the intact mesenteric arteries of rats in the WKY, SHR, OldWKY, and OldSHR groups using the Trizol reagent (invitrogen) according to the manufacturer’s protocol. Details of the protocol are available in the online Supplementary Methods. Briefly, the purified RNAs were subjected to cDNA synthesis, followed by adaptor ligation and enrichment, according to the manufacturer’s instructions on NEBNext^®^ Ultra™ RNA Library. After obtaining clean data and quality control, RNA sequencing and functional enrichment analysis were performed. For the differentially expressed RNAs (DEGs), the pathways were enriched based on DEGs through the KEGG pathway. The proteins of interest in the TXA2-TP-vasoconstriction pathways, including TXA2, TP, arrestin, actin, G protein-linked receptor kinases (Grk), and vasoconstriction-related genes, were specifically analyzed. Tools of Heatmapper (http://www2.heatmapper.ca/expression/) and Venn diagrams (https://bioinfogp.cnb.csic.es/tools/venny/index.html) were used. RNA sequencing data were uploaded to the Sequence Read Archive (SRA), and the BioProject accession number is PRJNA872876.

### Determination of protein expression

Total proteins or membrane proteins were extracted from the intact mesenteric artery of the rats in the WKY, SHR, OldWKY, and OldSHR groups using the liquid nitrogen grinding method or the kit method for extraction of membrane proteins (Invent, SM-005). The procedure was performed as previously reported (Cui et al., 2022). Details of the procedure are available in the Online Supplementary Methods.

### Determination of G actin/F actin

G/F-actin was determined by the analysis kit (G/F-actin *in vivo* analysis kit, Cat. No. bk037; cytoskeleton). The intact mesenteric artery was lysed in LAS2 buffer and the following procedure was in the online Supplementary Methods.

### Reagents and solutions

All reagents, which were of at least an analytical grade, used in the experiments were bought from the companies and the details are available in the online Supplementary Materials and Methods. Details of the solution preparation are available in the online Supplementary Materials and Methods.

### Statistical analysis

Results are expressed as mean ± S. D., and all data were normally distributed. Comparisons were performed using ANOVA or unpaired Student’s t tests, when appropriate, using GraphPad Prism 8.4.2 (GraphPad Software). *p* < 0.05 was considered statistically significant. For statistical analysis of RNA sequencing enriched in TXA2-TP related pathways, the criteria of fold change >1.5 and adjusted *p*-value < 0.05 were used.

## Results

### High K^+^-induced constriction of isolated mesenteric artery rings in rats

**FIGURE 1:**
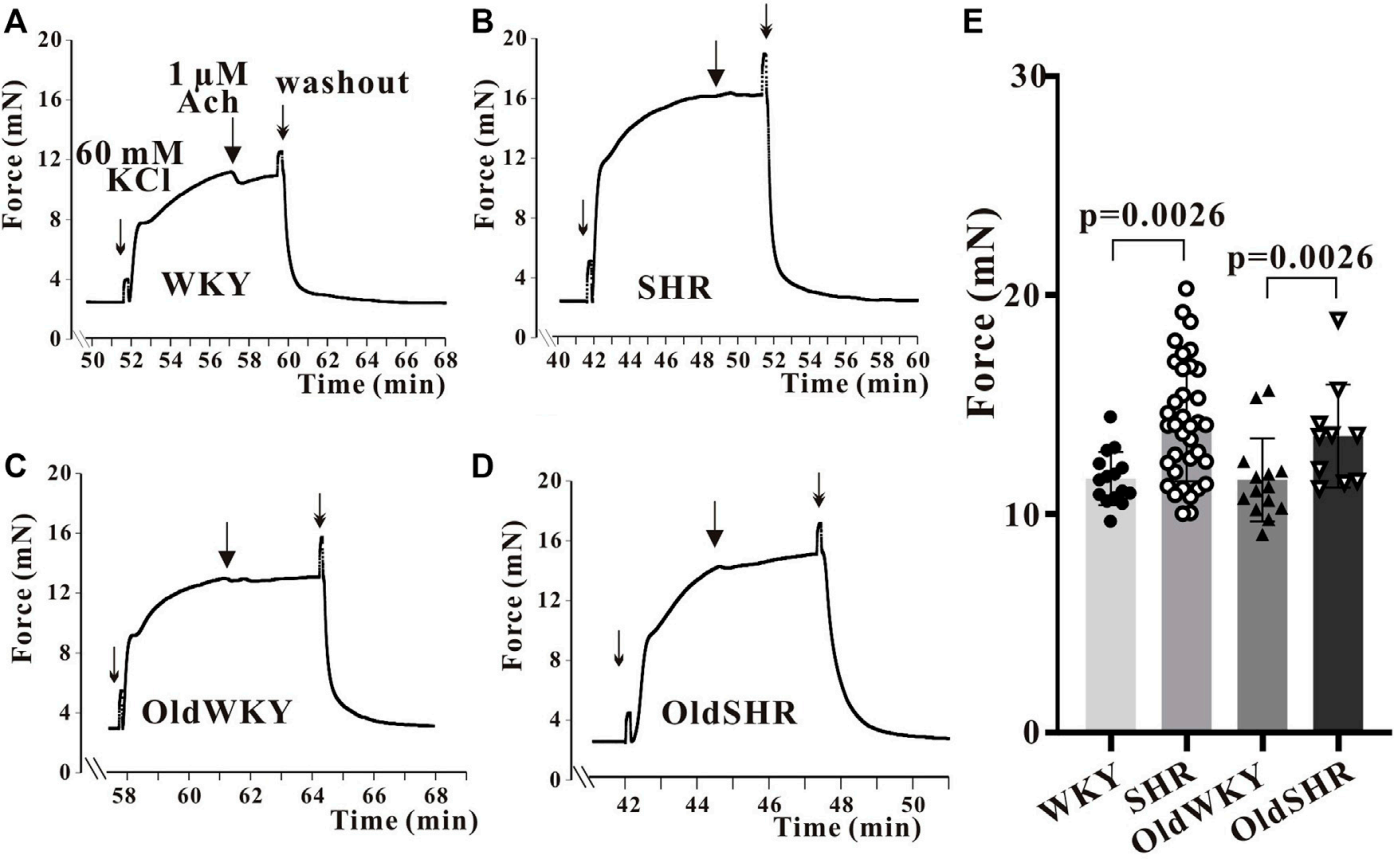
High K^+^ solution induced the maximum contraction of endothelium-denuded mesenteric artery rings. **(A–D)** Typical recordings showing vessel response to 60 mM K^+^ solution in the four groups. The X-coordinate time was recorded from the time when the vascular ring passed the activity test and was stabilized for another 30 min. **(E)** Summary data showed that mesenteric arteries are more sensitive to High K^+^ in hypertension. The mesenteric arteries of rats were obtained from 10 WKY rats, 16 SHR, 8 OldWKY rats, and 5 OldSHR, respectively.

The tertiary branches of the endothelium-denuded mesenteric arteries were used in the experiment. The maximal contractile response (Emax) induced by High K^+^ in the mesenteric artery was recorded and analyzed. As shown in Figure 1, Emax was significantly increased in SHR compared with WKY rats (*p* ＜0.01); Emax was significantly increased in OldSHR compared with OldWKY rats (*p* ＜0.01). This experiment showed that the mesenteric vessels from hypertension are more sensitive to High K^+^ in hypertension, which is consistent with other reports (Wei et al., 2021; Cui et al., 2022).

### Mesenteric vasoconstriction response induced by U-46619 was diminished remarkably in older rats and older hypertensive rats

**FIGURE 2:**
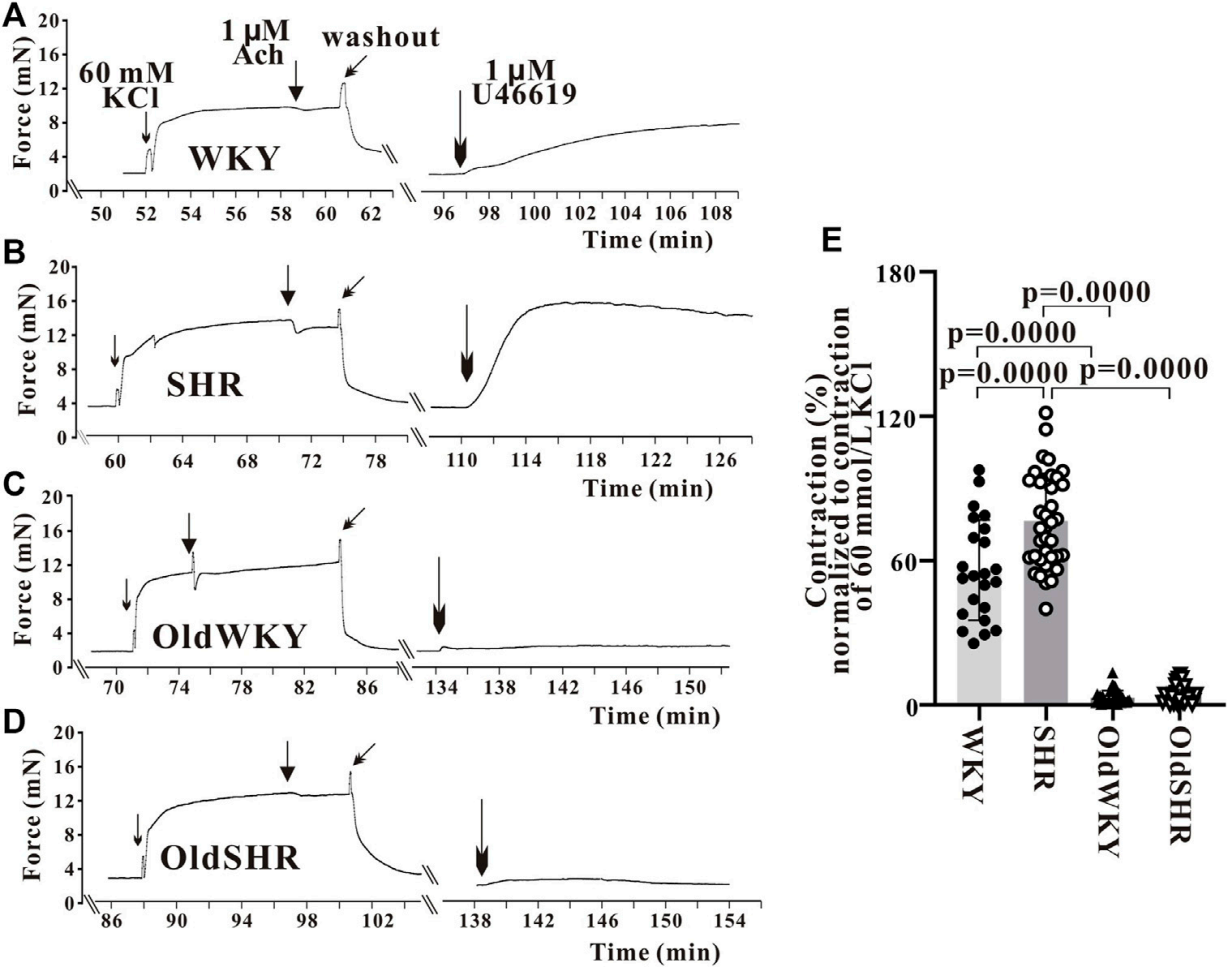
U-46619 induced the contraction of endothelium-denuded mesenteric artery rings. **(A–D)** Typical recordings showing vessel response to 1 μM U-46619 in the four groups. The X-coordinate time was recorded from the time when the vascular ring passed the activity test and was stabilized for another 30 min. **(E)** Summary data showing that mesenteric artery response to U-46619 was diminished remarkably in OldWKY and OldSHR groups. Mesenteric vessels were obtained from 16 WKY rats, 9 SHR rats, 17 OldWKY rats, and 18 OldSHR rats.

First, the endothelium-denuded mesenteric artery rings from WKY rats were used to determine the optimal concentration of U-46619-induced vasoconstriction. U-46619 (10^−10^–10^−5^ M) induced a concentration-dependent contraction of the mesenteric artery. A dose-response curve was obtained, and the concentration of U46619 causing 80% of Emax was 1 μM. Therefore, U-46619 at 1 μM was used in subsequent experiments. The mesenteric vessels were constricted with 1 μM U-46619 until Emax was achieved. The percentage of Emax caused by U-46619 relative to Emax caused by high K^+^ solution (%Emax) was calculated. The vasoconstriction induced by U-46619 was different from those of vascular responses induced by high K^+^ or PE, as previously reported (Wei et al., 2021). The response of mesenteric vessels to U-46619 was significantly increased in hypertension. However, surprisingly and contrary to expectations, response to U-46619 in OldWKY and OldSHR rats was minimal. As shown in Figure 2, the Emax of mesenteric artery induced by 1 μM U-46619 was significantly increased in SHR compared with WKY rats (*p <* 0.001). In OldWKY rats, Emax was, however, significantly decreased (*p* < 0.001). No significant difference in mesenteric artery constriction induced by U-46619 was found between OldWKY and OldSHR rats (*p* > 0.05). These data clearly showed that the mesenteric vasoconstriction induced by U-46619 was obstructed in older rats and older hypertensive rats.

### Vessels with impaired response to U-46619 in OldWKY and OldSHR rats had a strong contractile response to phenylephrine

**FIGURE 3:**
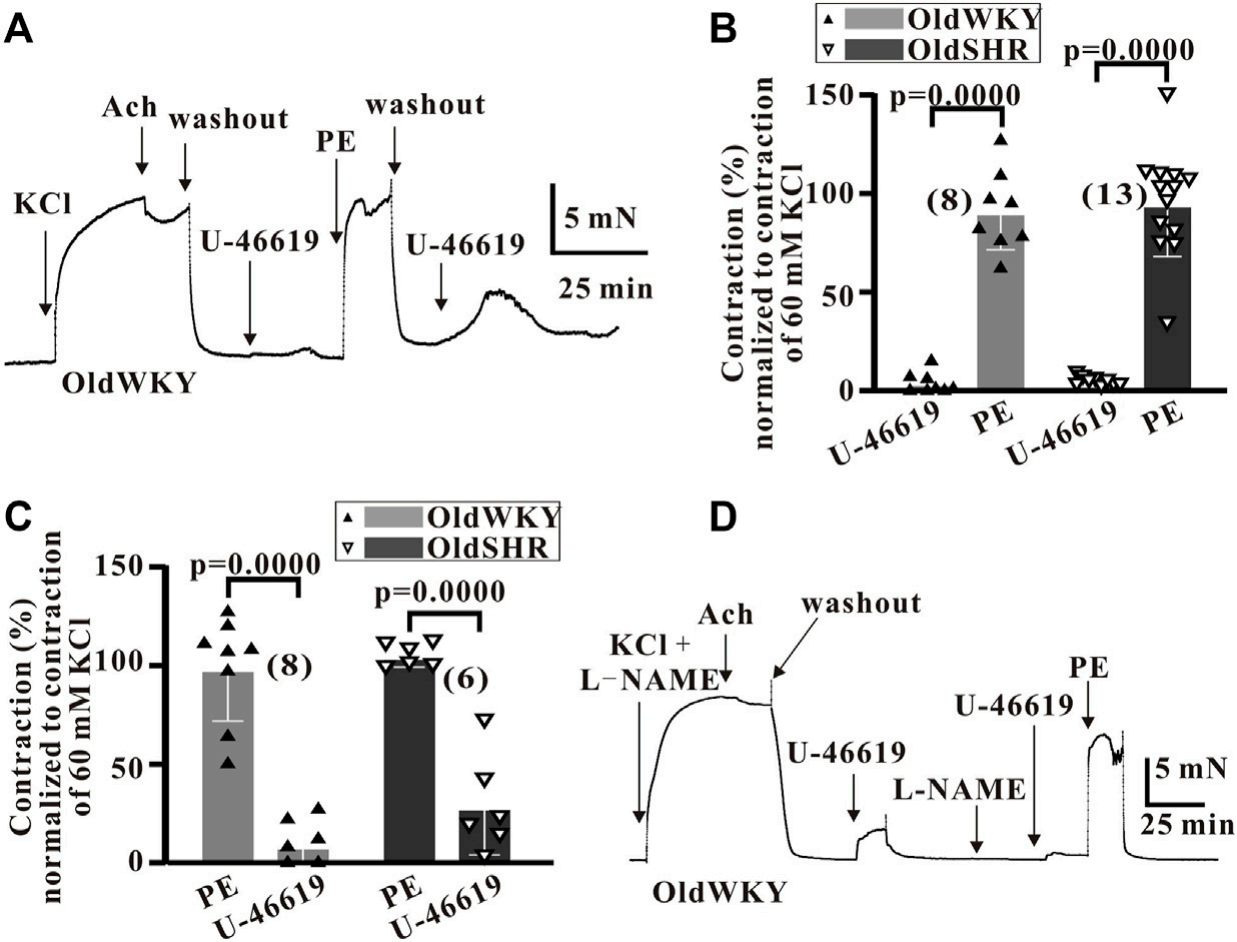
U-46619 and PE induced the contraction of endothelium-denuded mesenteric artery rings. **(A)** Typical recordings showing vessel response to 1 μM U-46619 and 5 μM in OldWKY rats, showing that the artery with impaired response to U-46619 had normal response to phenylephrine. **(B,C)** Summary data showing that vessels with reduced response to U-46619 in OldWKY and OldSHR rats had normal response to PE, showing similar results regardless of whether U-46619 or PE was used first. **(D)** Typical recordings showing vessel response to 1 μM U-46619 and 5 μM in OldWKY rats after the preconditioning of L-NAME, indicating that the artery with impaired response to U-46619 was not due to incomplete endothelial removal. Numbers inserted in the graphs represented the number of arteries used in the experiment. Arteries were obtained from 4–6 rats in each group.

To prove that the blood vessels used in the study had not been damaged by dissection or prior treatment, 5 μM phenylephrine (PE), an α1-adrenergic receptor (α1-AR) activator, was used as stimulant to observe the vascular reactivity (Figures 3A,B). As shown in Figure 3B, the %Emax of mesenteric vasoconstriction induced by U-46619, compared with that induced by High K^+^, were 4.75 ± 5.04 (*n* = 8) and 2.31 ± 4.29 (*n* = 13) in OldWKY and OldSHR rats, respectively. The relative contractions (%Emax) induced by PE were 91.17 ± 18.41 (*n* = 8) and 95.20 ± 26.16 (*n* = 13), respectively. These results suggested that in older rats and older hypertensive rats, U-46619 hardly elicited vasoconstriction, but the vessels had a strong contractile response to PE. Additionally, a similar set of experiments was conducted to explore whether PE might sensitize the receptor TP. In this study, the vascular responsiveness was observed by preconditioning with 5 μM PE and 1 μM U-46619. The results showed that vessels that normally responded to PE in OldWKY or OldSHR rats did not respond to U-46619, as similarly observed in the use of U-46619 prior to PE (Figure 3C). The results indicated that PE did not sensitize TP. All these results suggested that the prostaglandin signaling pathway associated with contraction in vascular smooth muscle was abnormally weakened, but the adrenergic α receptor pathway was not.

To rule out the possibility that the reduction in vasoconstriction induced by U-46619 was due to incomplete endothelial removal, the vessels were treated with 100 nM L-NAME in addition to vascular ring friction. The vasoreactivity was measured with 1 μM Ach, and the vessels with <20% vasodilation caused by ACh were used for the subsequent experiment. Thereafter, vascular reactivity was assessed by administering 1 μM U-46619 and/or 5 μM *p*E. Poor vascular response to U-46619 and strong response to PE in the vessels of OldWKY and OldSHR rats were recorded. This result suggested that the reduction in vasoconstriction response induced by U-46619 in OldWKY and OldSHR rats was not due to incomplete endothelial removal (typical Figure 3D).

### Differentially expressed genes in the TXA2-TP signaling pathway

**FIGURE 4:**
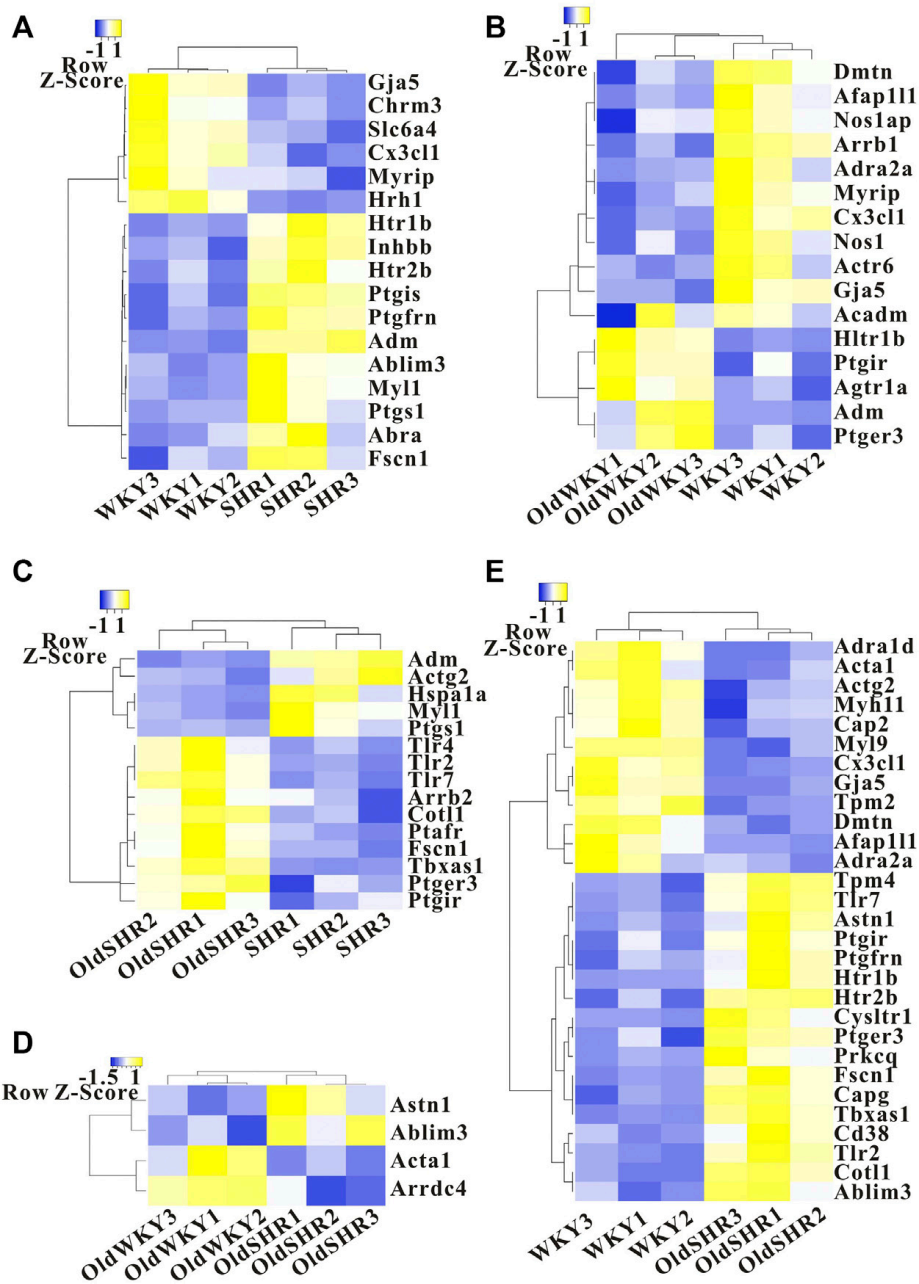
Heatmap showing differentially expressed genes in the TXA2-TP signaling pathway. **(A)** The SHR group was compared with the WKY group. **(B)** The OldWKY group was compared with the WKY group. **(C)** The OldSHR group was compared with the SHR group. **(D)** The OldSHR group was compared with the OldWKY group. **(E)** The OldSHR group was compared with the WKY group.

The study was intended to provide information for molecular biology validation experiments in the TXA2-TP-vasoconstriction signaling pathway. Similar number of DEGs caused by aging and hypertension (16 genes in aging and 17 genes in hypertension) were identified. The changes caused by hypertension were mainly upregulated gene expression, whereas the changes caused by aging were mainly downregulated gene expression (Figures 4A,B). The altered genes were different, and the common genes were *Myrip*, *Htr1b*, *Gja5*, *Cx3cl1*, and *Adm* (Supplementary Figure S1A), mainly reflecting in the common alteration in the myosin-interacting protein, hydroxytryptamine receptor, gap junction protein, C-X3-C motif chemokine ligand, and adrenomedullin. Changes in DEGs caused by aging factors under hypertensive conditions were more than those caused by hypertension factors under aging conditions (Figures 4C,D). Additionally, the related genes in the TXA2-TP signaling pathway of concern in this study did not change significantly. However, the common DEGs caused by aging factors and aging factors under hypertensive conditions mainly included Ptgir (prostagenin I2 receptor, IP) and Ptger3 (prostagenin E receptor, EP) (Figures 4A–E, Supplementary Figure S1B), suggesting changes in the prostaglandin signaling pathway in the older adult. We enriched the pathways based on DEGs through the KEGG pathway, but the prostaglandin signaling pathway could not be enriched (Supplementary Figure S2, the 30 most significant KEGG pathways were selected to construct a bar chart for display).

### Expression of TXA2-TP signaling pathway-related proteins

**FIGURE 5:**
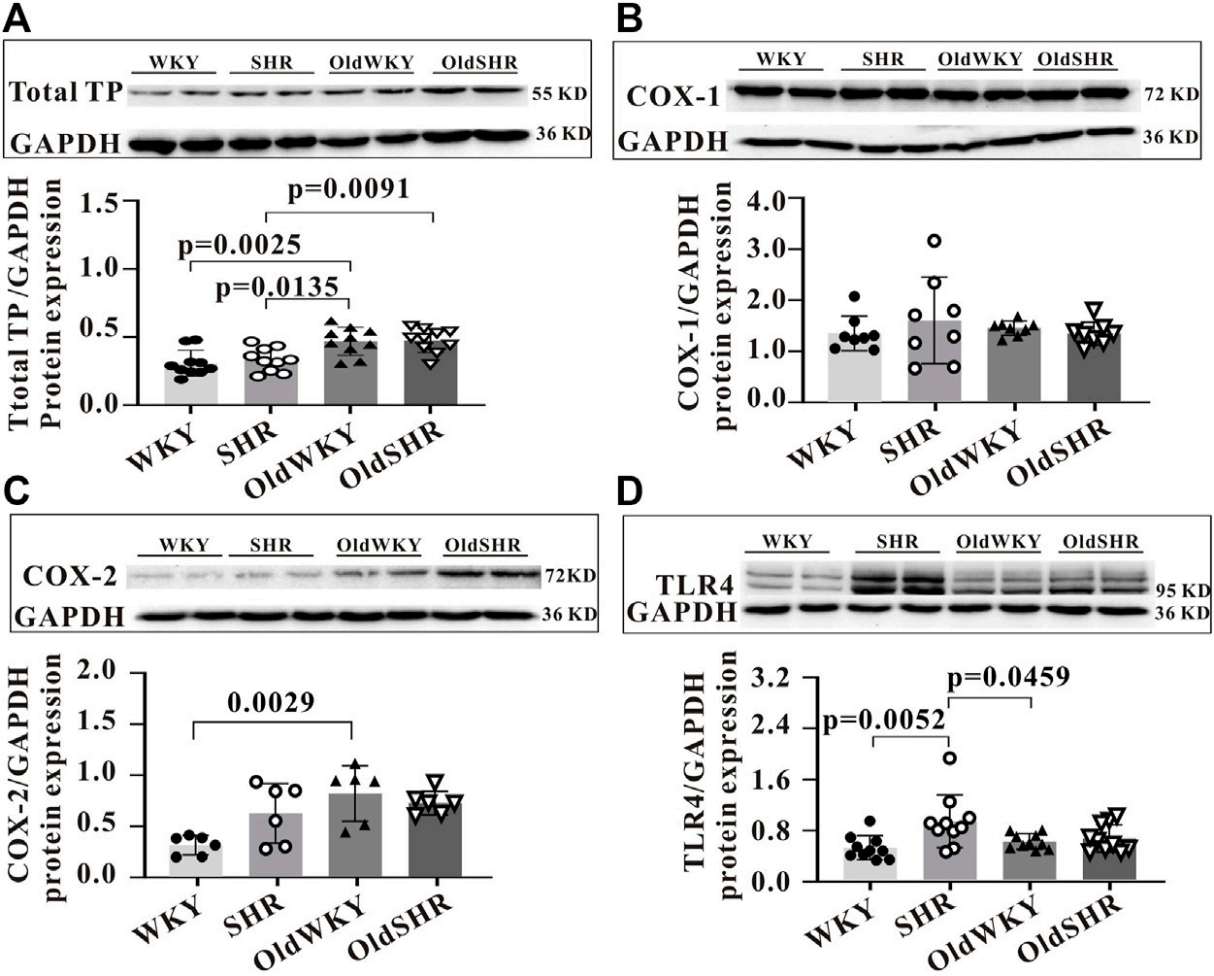
Expression of prostaglandin signaling pathway-related proteins. **(A)** Total TP protein expression, showing a significantly increased expression of TP in SHR, OldWKY, and OldSHR rats. **(B,C)** COX-1 and COX-2 protein expression, showing a significantly increased expression of COX-2 in SHR, OldWKY, and OldSHR rats. **(D)** TLR4 protein expression, showing a significantly increased expression of TLR4 in SHR and OldWKY rats. Intact mesentery arteries were collected from 5–6 rats in each group.

Although experiments on these DEGs did not provide much information on the TXA2-TP signaling pathway, we designed this experiment to further study the related proteins in this pathway, considering the significance of the proteins involved in this signaling pathway on vasomotor regulation. Endogenous arachidonic acids (AA) are mainly produced by phospholipid release from cell membrane. AA is metabolized by COX-1 or COX-2 to further form TXA2. The binding of TXA2 to specific receptors (TP) produces the basic physiological function of TXA2, such as vasoconstriction. Therefore, we checked the expression of the TXA2 signaling pathway-related proteins in vasoconstriction using the mesenteric arteries or serum from rats from the four groups. As shown in Figure 5A, the protein expression of total TP in the mesenteric arteries of OldWKY and OldSHR rats determined by western blot analysis was significantly increased. The expression of COX-1 and COX-2 was shown in Figures 5B,C, showing that COX-1 was not significantly altered, while COX-2 expression was significantly increased in OldWKY rats. As shown in Figure 5D, the expression of Toll-like receptor 4 (TLR4) in the mesenteric arteries of SHR was significantly increased, compared with that of WKY and OldWKY rats. Additionally, as shown in Supplementary Figure S3, findings from the ELISA showed that, compared with the WKY group, the other groups had no significant change in TXA2 expression in serum (*p* > 0.05). These results showed that the decreased vasoconstriction reactivity to U-46619 in OldWKY and OldSHR rats was inconsistent with the expression of TXA2, total TP, and TXA2 signaling pathway-related proteins.

### TP desensitization or endocytosis in OldWKY and OldSHR rats

**FIGURE 6:**
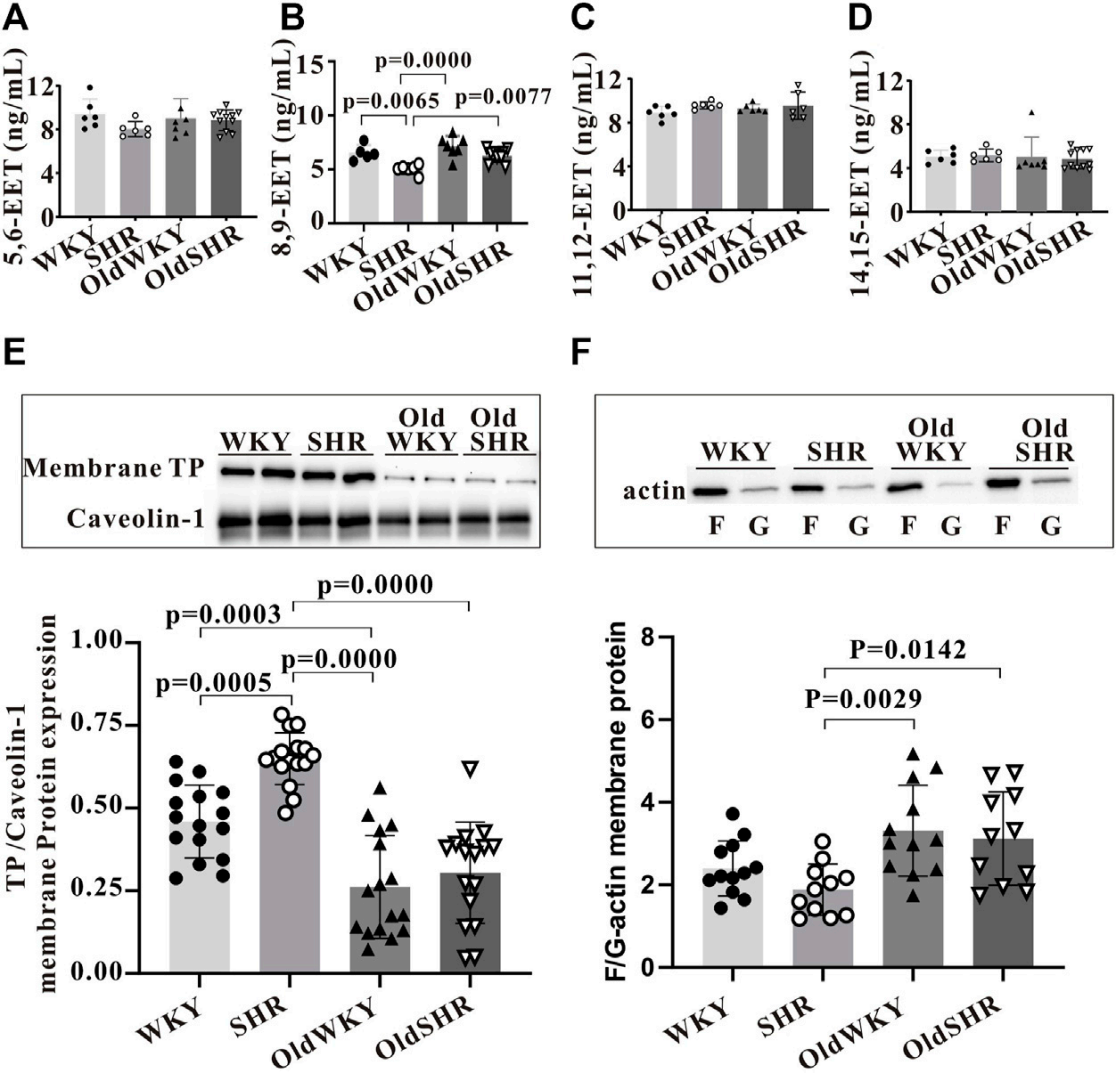
Expression of TP desensitization or endocytosis-related proteins. **(A–D)** Expression of EET subtypes in serum by ELISA. Summary data showing that 8, 9-EET serum levels were significantly higher in the OldWKY and OldSHR groups than in the SHR group (*p* < 0.001 and *p* < 0.01, respectively). **(E)** Western blot showing that expression of TP on the cell surface was significantly decreased in OldWKY or OldSHR rats (*p* < 0.001). **(F)** F/G-actin protein expression showing that F/G-actin ratio in mesenteric arteries of OldWKY or OldSHR rats was significantly increased, compared with SHR rats (*p* < 0.01 or *p* < 0.05). Intact mesentery arteries were collected from 5–11 rats in each group.

TXA2 or thromboxane A2 analogue (U-46619) binds to its dominant receptor, TP, and mediates vasoconstriction *via* the classical pathways (Gq-PLC-IP3/DAG-Ca2+/PKC or G12/13-RHO-MLC). The presence of endogenous TP antagonists, such as epoxyeicosatrienoic acids (EETs) (Behm et al., 2009), may cause receptor desensitization, resulting in decreased vascular response to U-46619. Therefore, the expression of total EETs in serum and mesenteric vascular tissue homogenates and the expression of EET subtypes in serum were determined (Figures 6A–D). However, no differences in the expression of total EETs in serum and mesenteric vascular tissue homogenates were observed among the four groups (data not shown). Notably, expression of some EET subtypes in serum was increased. As shown in Figure 6B, serum levels of 8,9-EET were significantly higher in OldWKY and OldSHR groups than those in the SHR group (*p <* 0.01), suggesting that attenuated vasoconstriction induced by U46619 may be associated with increased endogenous EET expression and resulting in receptor desensitization in OldWKY and OldSHR rats.

As stated, we confirmed that TP expression was significantly increased in the mesenteric arteries of SHR, OldWKY, and OldSHR rats in the above experiment. However, this finding reflected the expression of total TP protein in vascular tissues, rather than the expression of membrane surface receptors that directly interact with TXA2. Therefore, the expression of TP in membrane extracts of mesenteric artery tissues was detected. The results clearly showed that the expression of TP on the cell surface was significantly decreased in the OldWKY and OldSHR groups, compared with the WKY group, but significantly increased in the SHR group (Figure 6E).

Downregulation of cell surface receptors may be related to endocytosis and actin cytoskeleton during endocytosis (Laroche et al., 2005). Because our experiments showed an increase in endogenous TP stimulator EETs in older rats and older hypertensive rats, we wondered whether the actin-dependent arrestin-mediated mechanism (Laroche et al., 2005) was involved in TP endocytosis in OldWKY and OldSHR rats. Therefore, we hypothesized that the long-term increased endogenous stimulation of TP (e.g., 8, 9-EET) in older rats and older hypertensive rats, together with the remodeling of arrestins and actin cytoskeleton, led to the endocytosis of TP. We first detected the expression of β-arrestin 1 and β-arrestin 2. Unexpectedly, no significant changes of arrestins (Supplementary Figure S4) were observed in the OldWKY and OldSHR groups, which was inconsistent with the trend of vasoconstriction induced by U-46619. Thereafter, we examined the expression of F- and G-actin and found that F/G-actin ratio in the mesenteric arteries of SHR rats did not change significantly. However, the F/G-actin ratio in mesenteric arteries of OldWKY or OldSHR rats was significantly increased, compared with the ratio in SHR rats (Figure 6F, *p* < 0.01 or *p* < 0.05), suggesting that the increased F/G-actin contributed to actin-dependent TP endocytosis.

## Discussion

We report for the first time that the TXA2-TP prostaglandin signaling pathway is inhibited in older rats and older hypertensive rats, as demonstrated by the TXA-2 analog, U-46619, which significantly enhanced mesenteric vasoconstriction in SHR rats. However, interestingly and contrary to expectations, U-46619 almost did not cause mesenteric vasoconstriction in OldWKY and OldSHR rats. Mesenteric arteries with impaired response to U-46619 in OldWKY and OldSHR rats had strong responses to the adrenergic receptor agonist, PE. The de-endothelialized mesenteric artery experiment confirmed that the weak vascular response of U-46619 was unrelated to the vascular endothelium. Molecular biology results showed that the decreased expression of TP cell membrane proteins in mesenteric vessels and increased endogenous TP competitor, 8, 9-EET, in serum may be partly responsible for the decreased vascular reactivity to U-46619. The decreased expression of TP on the plasma membrane suggests that the receptor protein may escape from the plasma membrane and enter the cytoplasm by endocytosis, which may be related to actin cytoskeletal remodeling (Laroche et al., 2005; Smythe and Ayscough, 2006).

The main metabolites of endogenous AA were four types of epoxyarachidonic acid (EETs): 5, 6-; 8, 9-; 11, 12-; and 14, 15-EETs. The long-term increased endogenous stimulation of TP (such as EETs) may lead to the endocytosis or desensitization of TP. Our results showed significantly higher serum 8, 9-EET levels in OldWKY and OldSHR rats, suggesting that U46619-induced diminished vasoconstriction may be associated with increased endogenous EET expression. Moreover, AA may generate TXA2, cycloprostaglandins (PGI2), and prostaglandins (PGS) in the presence of COX-1 and COX-2. TXA2 binding with its specific receptor (TP) can cause thrombosis and vasoconstriction. Our study confirmed that the total protein expression of TP in mesenteric vessels in OldWKY and OldSHR rats significantly increased (Figure 5A), which was obviously contrary to the decreased vascular response to U-46619. Additionally, upregulated or unchanged expression of proteins was associated with TXA2-TP signaling, which is inconsistent with a decreased vascular response to U46619. The expression levels of total TP protein were significantly increased in older rats and older hypertensive rats, reflecting the upregulation of functional requirements for this pathway.

Importantly, our membrane protein experimental results clearly showed that TP expression on OldWKY and OldSHR cell surface was significantly decreased, which may be an important mechanism of impaired vascular response to U-46619 in older rats and older hypertensive rats. Changes in U-46619-induced vasoconstriction showed that the mesenteric vessels of OldWKY and OldSHR groups were not responsive to U-46619 but to α-AR activator PE, indicating a receptor-specific change. Actin consists of monomeric globular actin (G-actin) and polymeric fibrous actin (F-actin). G- and F-actin can be converted into each other to regulate the depolymerization or polymerization of actin cytoskeleton. Previous studies demonstrated that depolymerization of actin cytoskeleton blocked agonist-induced endocytosis of TP β subtype in HEK293 cells, suggesting that TP endocytosis requires active remodeling of actin cytoskeleton (Laroche et al., 2005). Consistent with this result, our study confirmed the contribution of the increase in F/G ratio in OldWKY and OldSHR rats (Figure 6F). Taken together, our results suggested that the long-term increase in endogenous EET in older rats and older hypertensive rats, coupled with an increase in F/G-actin, resulted in endocytosis of TP.

In addition to the mechanism of TP desensitization discussed above, the DEGs were included in biological pathways for comprehensive analysis to better understand the biological process of TXA2-TP-vasocontaction. The degree of influence and rule of functional variation on biological pathways were analyzed for future studies. We enriched the pathways using DEGs through the KEGG pathway, but the prostaglandin signaling pathway could not be enriched (Supplementary Figure S2). We further analyzed DEGs related to prostaglandin receptors, smooth muscle contractile proteins, actin cytoskeletal remodeling, and vasoconstriction-related biological process signaling pathways (Figure 4). Similar number of DEGs caused by aging and hypertension were identified, and the common alteration mainly reflected in the myosin-interacting protein, hydroxytryptamine receptor, gap junction protein, C-X3-C motif chemokine ligand, and adrenomedullin. Importantly, the common DEGs caused by aging factors and aging factors under hypertensive conditions included Ptgir and Ptger3 (encodes prostaglandin I2 receptor and prostaglandin E receptor 3, respectively), suggesting changes in prostaglandin signaling pathways under aging pathological conditions. Ptgir is predicted to enable prostacyclin receptor activity, which is involved in the adenylate cyclase-activating GPCR signaling pathway, negative regulation of platelet-derived growth factor receptor signaling pathway, and negative regulation of smooth muscle cell proliferation. Ptger3 enables prostaglandin E receptor activity and acts upstream of or within several processes, including GPCR signaling pathway, bicarbonate transport, and fever generation. Combined with our findings of changes in TP receptors, F/G-actin, and DEGs, our study confirmed an age-related dysregulation of prostaglandin signaling. These changes had no significant differences between OldWKY and OldSHR rats, suggesting that the aging factor played a key role in this damaged vasoconstriction and hypertension did not exacerbate the damage.

Arrestins are adaptor proteins that function to regulate GPCR signaling and trafficking. Arrestin-mediated endocytosis of GPCRs requires the coordinated interaction between β-arrestins and clathrin, adaptor protein 2, and phosphoinositides, such as PIP2/PIP3 (Moore et al., 2007; Shenoy and Lefkowitz, 2011; Gurevich and Gurevich, 2015). Studies demonstrated that arrestins play essential roles not only in GPCR desensitization (Carman and Benovic, 1998) but also in receptor endocytosis (Gurevich and Gurevich, 2006). In consideration of the importance of arrestins in receptor desensitization and endocytosis, we performed special assays and found that β-arrestin 1 and β-arrestin 2 seemed not to play a major role in TP desensitization in OldWKY and OldSHR rats. In our study, DEG analysis showed that expression of the gene encoding β -Arrestin 1 was significantly downregulated in older rats (Figure 4B), whereas the expression of the gene encoding β-arrestin 2 was significantly upregulated in older hypertensive rats (Figure 4C). Protein expression analysis revealed that β -Arrestin 2 increased under hypertensive conditions (Supplementary Figure S4). The significance of these changes will be further studied in the future.

Dysregulation of TP might result in agonist hypersensitivity and hyper-responsiveness. Hinton et al. (2007) examined TP localization and kinetics following hypoxia to determine the mechanism of hypoxia-induced TXA2 hypersensitivity. They demonstrated that TP is desensitized in neonatal pulmonary circulation, usually through PKA-mediated phosphorylation, resulting in decreased receptor-ligand affinity and reduced receptor-ligand binding to Gq. This protection is lost after hypoxic exposure. Using site-directed mutational analysis, Santhosh et al. (2014) examined the role of PKA-accessible serine residues in determining TP affinity. They found that, in hypoxic pulmonary hypertension, loss of site-specific phosphorylation of the TP receptor caused agonist hyper-responsiveness. Feng et al. (2011) reported the contractile response of human coronary microvasculature to TXA2 before and after cardioplegia, followed by reperfusion (CP/Rep). They concluded that CP/Rep decreased the contractile response of human coronary arterioles to U-46619 soon after cardiac operations. However, they were unable to find the exact cause of the decreased vascular response to U46619. They reported that the contractile response to TXA2 required activation of TP and PLC but not PKC-α. They suggested that a period of intraoperative hypothermic ischemia and reperfusion may modify the functional state of TXA2 receptor–dependent signaling rather than the steady-state levels of their proteins. Cupitra et al. (2020) reported the influence of ageing on vascular reactivity and receptor expression in rabbit aorta. They found that ageing caused a decrease in the sensitivity of the aorta to KCl, U-46619, and isoproterenol, and an increase in the adrenergic response with aging. They indicated that the vascular reactivity to the TXA2 analogue showed a marked difference in contraction between young and old rabbits and between adult and old rabbits, suggesting that sensitivity to prostanoids remains until 6 months of development. However, the sensitivity deteriorates in old age in rabbits. Their finding indicated a significantly increased expression of α1 and β2 receptors in the intima and adventitia and increased expression of TP only in the adventitia. In contrast, our previous study (Wei et al., 2021) demonstrated a significant increase in the mesenteric artery contraction induced by PE in OldSHR. The mechanism is not consistent with the downregulation of α1-adrenergic receptor (α1- AR) expression but related to the increased expression of the downstream signaling molecule, PKC. PKC phosphorylates PKC-potentiated phosphatase inhibitor protein, CPI-17, which inhibits the activity of myosin phosphatase target subunit 1 of MLC phosphatase. Therefore, MLC20 phosphorylation level was increased, and vascular smooth muscle contraction was enhanced.

Our results also showed that the reactivity of both OldWKY and OldSHR rats to α1-AR was increased, and the reactivity to KCl was increased in the SHR and OldSHR groups. One of our previous studies (Cui et al., 2022) showed that vasodilation by NO in OldSHR rats was decreased, which significantly correlated with the reduction in the PKG-mediated effect. Our present study found that the TXA2 analog, U-46619, significantly enhanced mesenteric vasoconstriction in SHR rats but hardly caused mesenteric vasoconstriction in OldWKY and OldSHR rats. Consistent with changes in vasoconstrictor function, TP expression on the cell membrane was increased in SHR rats but decreased in the OldWKY and OldSHR groups. Our present findings indicated that the sensitivity to prostaglandins increased in hypertension, but the sensitivity deteriorated with age. This age-induced change could not be reversed even in hypertension. Additionally, findings from some reports were inconsistent with those from this study. For example, a study reported no difference in the expression of TP receptor in endothelial cells between SHR and WKY rats at 36–72 weeks (Tang and Vanhoutte, 2008). The aorta of male Fischer F344 rats at 6 and 22 months of age showed no difference in response to U-46619 stimulation (Shams et al., 1990), and the cerebral arterioles of rats showed no difference in response to the same agonist (Mayhan et al., 1990). The differences in the results from different laboratories could be due to the different animals and blood vessels used.

Agonist-induced desensitization is usually found to be associated with receptor phosphorylation by various protein kinases. The current theory is that the phosphorylation prevents proper docking of G proteins to the receptor, thus preventing or reducing signal transmission (Biringer, 2021). Wang et al. (1998) suggested a potential source for the initial phosphorylation. They reported that a cGMP-dependent G kinase (PKG) phosphorylated the TP receptor, which in turn inhibited IP3 release. Additionally, they showed that cAMP-dependent A kinase (PKA) was not involved. GPCR desensitization is thought to involve either cross-talk/heterologous desensitization among different signaling systems or agonist-induced/homologous desensitization terminating or modulating the response to the receptor. A study by Kelley-Hickie et al. (2007) on the mechanism of desensitization of signaling by TPα, suggested a mechanism whereby U-46619-induced diacylglycerol-regulated protein kinase C (PKC) phosphorylation of ^145^Ser partially and transiently impaired TPα signaling, whereas PKG- and PKC-phosphorylation at both ^331^Ser and ^337^Thr, respectively, effectively terminated its signaling. However, unlike in TPβ, G protein linked receptor kinases (GRK)/β-arrestins had no role in TPα homologous desensitization.

Based on the results of the expression of TP membrane receptors and F/G-actin in our study, we consider the changes insufficient to explain the phenomenon that the vessels of OldWKY and OldSHR rats barely respond to U-46619. Therefore, we speculated that the loss of vascular reactivity of U-46619 in older rats and older hypertensive rats might be related to TP phosphorylation. Unfortunately, no suitable TP phosphorylation-specific antibodies are available, and the reported cell-level TP phosphorylation experiments are not applicable to animals. Therefore, TP phosphorylation was not examined in our study. We previously reported (Wei et al., 2021) that the mRNA and protein expression of PKC in rat mesenteric artery obtained from SHR, OldWKY, and OldSHR rats increased significantly. The expressions of PKCγ and PKCζ were significantly increased in OldSHR rats, resulting in great changes in vascular function. Additionally, we reported that mRNA and protein expression of PKG was significantly elevated in SHR and OldSHR rats (Cui et al., 2022). Although we have not been able to determine PKA, our results showed that Adcy3, the main encoding gene of adenylyl cyclase, was upregulated during aging and downregulated in SHR and OldSHR rats (Cui et al., 2022). However, all these changes in PKC, PKG, or perhaps PKA that may lead to receptor phosphorylation seem to be inconsistent with the vasoconstriction changes induced by U-46619 in OldWKY and OldSHR rats. Growing evidence indicated that contraction was regulated by RhoA-Rho kinase (ROCKs). ROCK-1 expression had a large increase in hypertension, while ROCK-1 expression decreased with age. ROCK-2 expression increased significantly with age (Wei et al., 2021). However, the relationship between ROCKs and TP has not been discovered and reported, and whether ROCKs may lead to TP phosphorylation remains undetermined.

In summary, this study demonstrated for the first time the decreased vascular reactivity to U-46619 in OldWKY and OldSHR rats, indicating that the TXA2-TP prostaglandin signaling pathway was diminished in older adults. The evidence presented here complements the mechanisms underlying changes in vascular TXA2-TP signaling during aging-related vascular remodeling. The findings may provide a scientific rationale for developing novel therapeutic strategies for the treatment of older patients.

## Data Availability

The datasets presented in this study can be found in online repositories. The names of the repository/repositories and accession number(s) can be found below: https://www.ncbi.nlm.nih.gov/bioproject/PRJNA872876.
