# Supplementary material for: Thromboxane-induced contractile response of mesenteric arterioles is diminished in the older rats and the older hypertensive rats
Source: Front Pharmacol. 2022 Oct 12;13:1019511. doi: 10.3389/fphar.2022.1019511 (PMC9602936; doi:10.3389/fphar.2022.1019511)
Supplement: Supplementary file 1 [file DataSheet1.PDF]

# Thromboxane-Induced Contractile Response of Mesenteric Arterioles Is Diminished in the Older Rats and the Older Hypertensive Rats

Min Zhang†, MD, Chunshu Li†, MD, Chunxia He, MD†, Yiqin Cui, MD, Yuan Li, MD, Ying Ma, MD, Jun Cheng, MD, Jing Wen, MD, Pengyun Li, MD, PhD, Yan Yang, MD *

Key Lab of Medical Electrophysiology of Ministry of Education and Medical Electrophysiological Key Lab of Sichuan Province, Collaborative Innovation Center for Prevention and Treatment of Cardiovascular Disease, Institute of Cardiovascular Research, Southwest Medical University, Luzhou, Sichuan 646000, China

## Online Supplement

Files in this online supplement: Materials and methods, Supplementary table 1, Supplemental Figure 1, Supplemental Figure 2, Supplemental Figure 3, and Supplemental Figure 4.

# Supplemental Materials and methods:

**Table 1. Information of rats used in the study**

| | WKY | SHR | OldWKY | OldSHR |
|---|---|---|---|---|
| **Number of rats** | 25 | 30 | 25 | 22 |
| **Age (months)** | 4 | 4 | 18 | 18 |
| **body weight(g)** | 325.6 ± 23.4 | 305.9 ± 20.8 | $456.2 \pm 32.5^{\Delta\Delta\Delta\Delta\#\#\#\#}$ | $360.3 \pm 19.2^{\&\&\&\&\$\$\$\$}$ |
| **Systolic (mmHg)** | 111.1± 14.3 | $187.5 \pm 23.9^{****}$ | $130.7 \pm 16.8^{\Delta\Delta\#\#\#\#}$ | $200.7 \pm 26.5^{\$\$\$\$}$ |
| **Diastolic (mmHg)** | 80.6 ± 11.5 | $142.2 \pm 18.1^{****}$ | $86.7 \pm 14.6^{\#\#\#\#}$ | $145.9 \pm 22.1^{\$\$\$\$}$ |
| **Heart rate (bmp)** | 319.6 ± 41.4 | $358.4 \pm 52.9^{*}$ | $284.1 \pm 50.6^{\#\#\#\#}$ | $322.0 \pm 47.3^{\&\$}$ |
| **Blood glucose** | 5.39 ± 0.53 | 4.98 ± 0.46 | $5.95 \pm 0.50^{\Delta\#\#\#\#}$ | $5.07 \pm 0.73^{\$\$\$}$ |

*P < 0.05, SHR *vs.* WKY rat group; $^{\Delta}$P < 0.05, OldWKY *vs.* WKY group; $^{\#}$P < 0.05, OldWKY *vs.* SHR group; $^{\&}$P < 0.05, OldSHR *vs.* SHR group; $^{\$}$P < 0.05 OldSHR *vs.* OldWKY group.

**Preparation of mesenteric vascular rings**

Rats were anesthetized with isoflurane using a specific isoflurane evaporator and blood pressure of rats was measured by femoral artery cannula, and then the rats were euthanized. The tertiary branches of mesentery arteries were excised and placed in cooled Tyrode solution. The outer connective tissue was gently removed and the mesenteric artery was cut into 2~ 3 mm sections and a stainless steel wire was slided gently into the lumen of the blood vessel to remove the endothelium. Tyrode solution was added into the chamber and constantly gassed with 95% oxygen, and the temperature was maintained at 37 ±0.5℃ and pH at 7.4. The prepared vascular ring was mounted in a DMT myograph system and was stretched in a step-wise manner to set to a basal tension of 2 to 4 mN. The artery ring was balanced for 60 min and the chamber solution was exchanged once every 20 minutes. High $K^+$ (60 mM $K^+$ solution) was used to detect vascular reactivity 60 minutes later. After the vessels reached maximum contraction, 1 µM acetylcholine (Ach) was used to detect endothelial activity. If the vasodilation caused by Ach was less than 20%, the vessels were considered to have no endothelial residue and the vessel can be used for subsequent experiments. High $K^+$ was repeated to make the blood vessels contract twice, and the difference between the two contractions was calculated. If the difference was less than 10%, the maximum contraction caused by High $K^+$ was considered to be stable. Then, the bath was washed with Tyrode's solution, and the follow-up experiment was conducted after 30 min stability.

**RNA sequencing and functional enrichment analysis**

Total RNA was isolated from mesentery artery of WKY, SHR, OldWKY and OldSHR groups using the manufacturer's protocol of Trizol (invitrogen). RNA purity was determined using ND-1000 Nanodrop. The A260:A280 ratio of each RNA sample was above 1.8, and the A260:A230 ratio was above 2.0. RNA integrity was assessed using Agilent 2200 TapeStation (Agilent Technologies, USA) to ensure that RIN in each sample was above 7.0. rRNAs were removed from total RNA using the epicentreriboo-zero rRNA removal kit (illumina, USA) and fragmented to approximately 200 bp. The purified RNAs were subjected to first strand and second strand cDNA synthesis following by adaptor ligation and enrichment with a low-cycle according to instructions of NEBNext® Ultra™ RNA Library. Agilent 2200 TapeStation and Qubit®2.0 (Life Technologies, USA) were used to evaluate the purified library products. After dilution to $10^{-9}$ mol/L, clusters were generated in situ on the pair-end flow cell followed by sequencing (2×150 bp) HiSeq3000. Quality analysis of the raw data was performed to assess the suitability of the sequencing data for bioinformatics analysis. According to the analysis results of the original data, the data were filtered, the joint sequence was removed, the contaminated part was removed, and then the sequence containing too many low quality bases was removed, and finally, Cleandata was obtained. Perform quality statistics on Cleandata to ensure bioinformatics analysis requirements. Differential expression was assessed by DEseq using read counts as input. Differentially expressed genes were chosen according to the criteria of fold change > 1.5 and adjusted p-value < 0.05. The differentially expressed RNAs and the enrichment in TXA2-TP pathway-vasoconstriction were mapped using Heatmapper (http://www2.heatmapper.ca/expression/) and Venn diagrams with the tool on https://bioinfogp.cnb.csic.es/tools/venny/index.html.

**Determination of Protein Expression**

Total protein was extracted from the intact mesenteric artery of the WKY, SHR, OldWKY, and OldSHR using liquid nitrogen grinding method. Briefly, the vascular tissue was adequately grinded by liquid nitrogen method and then added proper amount of lysis buffer lysate containing of protease inhibitor according to the weight of the tissue. Placed tissue on ice for 30 min and grinded the total protein of vascular smooth muscle, the supernatant was obtained after 15 min centrifugation at 4 ℃ and 12000 r/min. BCA protein assay kit was used to determine the protein concentration. Samples were isolated on 10% SDS-PAGE and transferred to PVDF membrane (400 mA electric current for 1 min per 1 kDa).Then the PVDF membrane was blocked with 5% non-fat powdered milk (0.1% TBST) for 2 h at room temperature, and washed with 0.1% TBST 3 times for 10 minutes once. Then the membrane was incubated with TP antibody (Abcam, ab50358, 1:800), COX-1 antibody (Cell signaling, #4841, 1:1000), COX-2 antibody (Cell signaling, #4842, 1:800), TLR4 antibody (Novus, HTA125, 1:800), and GAPDH (Beijing biyuntian, AG019-1, 1:2000) overnight at 4 ℃. The membrane was washed 3 times with 0.1% TBST for 5 minutes and then incubated with the secondary antibody (Goat anti-Rabbit/Mouse IgG (H+L)-HRP ) at room temperature for 2 h. Images were obtained and quantified using Quantity One software (J 1.49v, Bio-Rad, CA, USA). The relative expression of the target protein is expressed by the ratio of the target protein to GAPDH.

To obtain the mesenteric arterial membrane protein, the kit method for extraction of membrane protein was adopted (Invent, SM-005). Briefly, the mesenteric artery tissue samples were added to different buffers and centrifuged at different speeds to obtain different cell components. The membrane protein precipitate was suspended with 0.1% SDS containing protease inhibitor and denaturated at 99℃ for 10 min. Membrane protein was separated by 10% SDS-PAGE gel electrophoresis and

transferred to PVDF membrane. After blocking the membrane with protein free rapid blocking buffer, the membrane was incubate with anti-Thromboxane A2 receptor antibody (abcam, ab134959, 1:1000), anti-beta Arrestin 1 antibody (abcam,ab32099, 1:1000), anti-beta Arrestin 2 antibody (abcam,ab206972, 1:1000), Caveolin-1 (cell signaling technology, 3267, 1:1000) at 4℃ overnight. Images were obtained after the secondary antibodies (BBI, D110058) and data were analyzed using Image J 1.49v and Graphpad prism 8.4.2.

**Determination of G actin /F actin**

G/F-actin was determined by the analysis kit (G/F-actin in vivo analysis kit, Cat. No. bk037; Cytoskeleton Inc.). The intact mesenteric artery was lysed in LAS2 buffer at 37 °C for 10 minutes. The lysates was centrifuged at 350xg at room temperature for 10 minutes, and the supernatant was centrifuged at 100000xg in an ultracentrifuge preheated to 37 °C for 1 hour to granulate F-actin and retain G-actin in the supernatant. The supernatant was collected as the G-actin component solution. F-actin depolymerization buffer with the same volume as that before centrifugation was added to the granulated F-actin, and incubate it on ice for 1h to depolymerize F-actin to obtain the F-actin component solution. Finally, 5xSDS sample buffer was added into the G-actin component solution and the F-actin component solution by 4:1 and mixed evenly. The samples were stored at - 20 °C for SDS-PAGE and Western blot analysis.

Proteins were isolated using the Omni-EasyTM one-step PAGE Gel Rapid Preparation kit (10%). After transfer, the membrane was blocked with protein free rapid blocking buffer at room temperature for 30 minutes and then was incubated with primary antibody（anti actin antibody 1:1000，Cat. No. bk037; cytoskeleton)）for 1 h at room temperature. After cleaning, the membrane was subjected to chemiluminescence detection of actin (43 kDa). Finally, Western blot analysis was performed with ImageJ to calculate the ratio of F-actin / G-actin.

**Reagents and solutions**

9,11-dideoxy-9α,11α-methanoepoxyprostaglandin F2α (U-46619, CAS:56985-40-1, Sigma-Aldrich Co.), phenylephrine hydrochloride (PE, No. HY- B0471, MCE Co.), acetylcholine (Ach, CAS: 60-31-1, Sigma-Aldrich Co.), and NG-nitro-L-arginine methyl ester (L-NAME, No.S0006, Beyotime Co.) were bought from the reagent companies. All Reagents used in the experiments were at least analytical grade. Tyrode solution contained (mmol/L): NaCl 127, KCl 5.9, $CaCl_2$ 2.4, $MgCl_2$, Glucose 12, HEPES 10. High $K^+$ solution (60 mmol/L KCl solution) was prepared by using equimolar KCl to replace NaCl in Tyrode solution. Tyrode solution and high $K^+$ solution were adjusted pH to 7.4 with NaOH.

## Results:

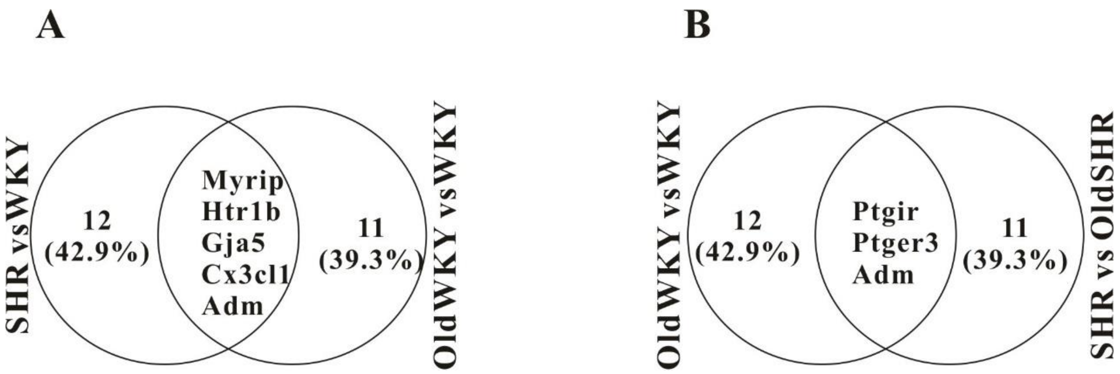


**Supplemental Figure 1. Venn diagram of differentially expressed genes TXA2-TP signaling pathway.** (A) Venn diagram showing the differentially expressed the common genes caused by aging factor and hypertension factor; (B) Venn diagram showing the common differentially expressed genes caused by the aging factor and the aging factor under hypertension.

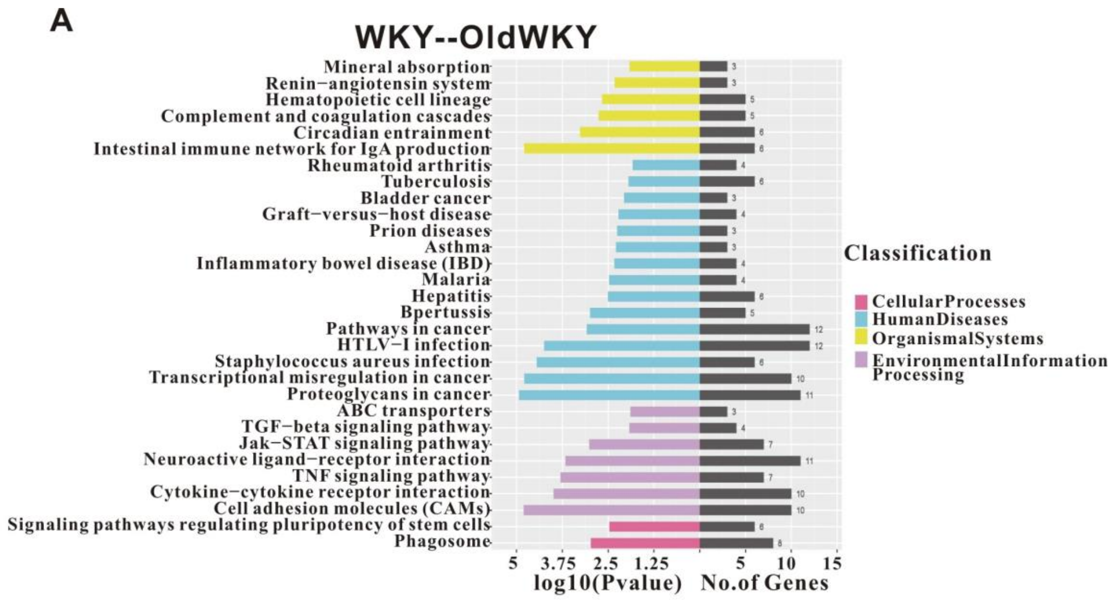
A
WKY--OldWKY
Mineral absorption
Renin−angiotensin system
Hematopoietic cell lineage
Complement and coagulation cascades
Circadian entrainment
Intestinal immune network for IgA production
Rheumatoid arthritis
Tuberculosis
Bladder cancer
Graft−versus−host disease
Prion diseases
Asthma
Inflammatory bowel disease (IBD)
Malaria
Hepatitis
Bpertussis
Pathways in cancer
HTLV−I infection
Staphylococcus aureus infection
Transcriptional misregulation in cancer
Proteoglycans in cancer
ABC transporters
TGF−beta signaling pathway
Jak−STAT signaling pathway
Neuroactive ligand−receptor interaction
TNF signaling pathway
Cytokine−cytokine receptor interaction
Cell adhesion molecules (CAMs)
Signaling pathways regulating pluripotency of stem cells
Phagosome
5 3.75 2.5 1.25
5 10 15
log10(Pvalue)
No.of Genes
Classification
CellularProcesses
HumanDiseases
OrganismalSystems
EnvironmentalInformation Processing

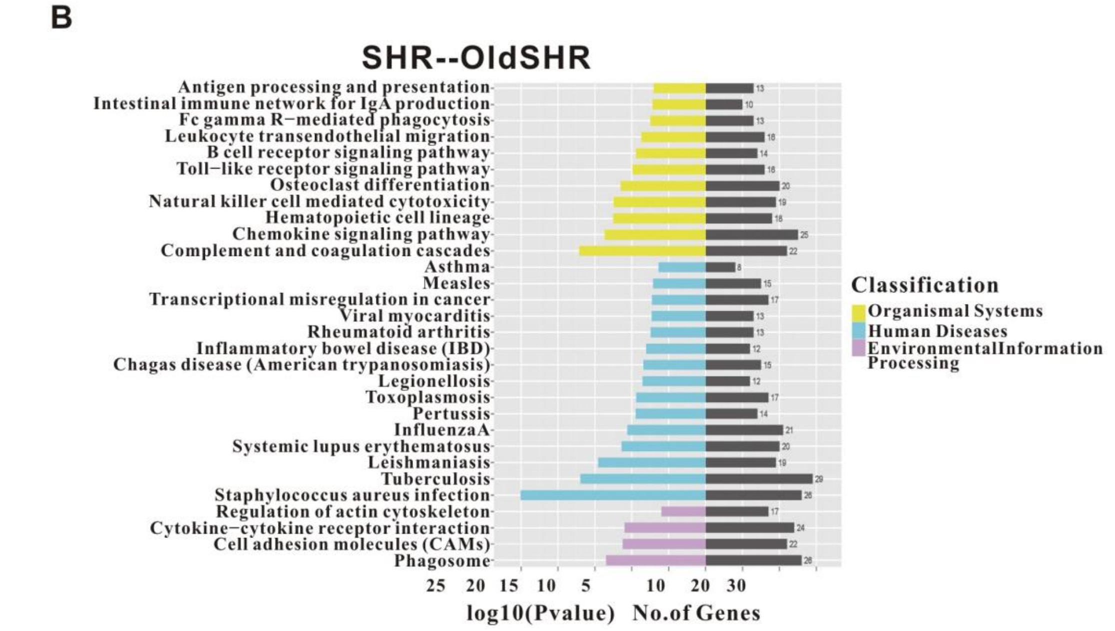
B
SHR--OldSHR
Antigen processing and presentation
Intestinal immune network for IgA production
Fc gamma R−mediated phagocytosis
Leukocyte transendothelial migration
B cell receptor signaling pathway
Toll−like receptor signaling pathway
Osteoclast differentiation
Natural killer cell mediated cytotoxicity
Hematopoietic cell lineage
Chemokine signaling pathway
Complement and coagulation cascades
Asthma
Measles
Transcriptional misregulation in cancer
Viral myocarditis
Rheumatoid arthritis
Inflammatory bowel disease (IBD)
Chagas disease (American trypanosomiasis)
Legionellosis
Toxoplasmosis
Pertussis
InfluenzaA
Systemic lupus erythematosus
Leishmaniasis
Tuberculosis
Staphylococcus aureus infection
Regulation of actin cytoskeleton
Cytokine−cytokine receptor interaction
Cell adhesion molecules (CAMs)
Phagosome
25 20 15 10 5
10 20 30
log10(Pvalue)
No.of Genes
Classification
Organismal Systems
Human Diseases
EnvironmentalInformation Processing

C

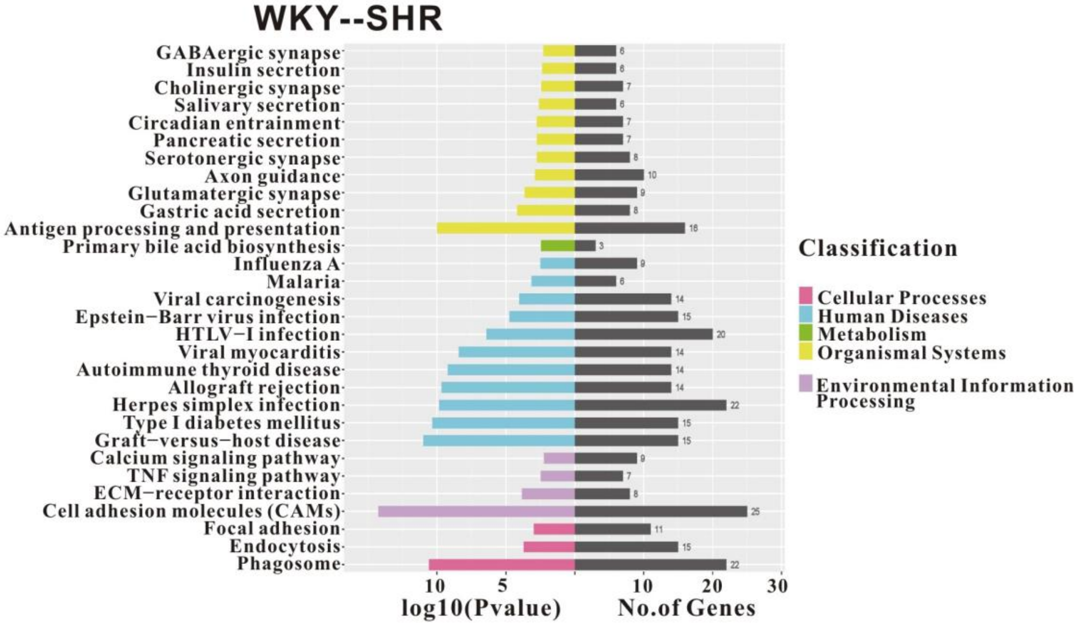


D

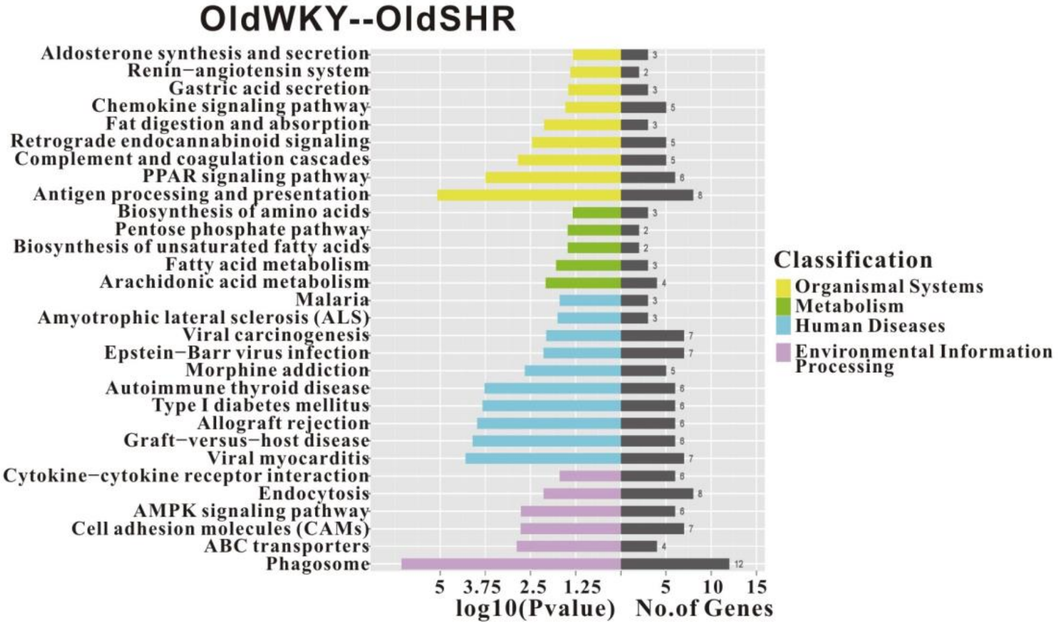


**Supplemental Figure 2. Multiple pathways were enriched based on the differentially expressed RNAs (DEGs) through KEGG pathway.** (A) OldWKY compared with WKY. (B) SHR-compared with OldSHR. (C) SHR compared with WKY. (D) OldWKY compared with OldSHR. The most significant 30 KEGG pathways were selected to draw a bar chart for display.

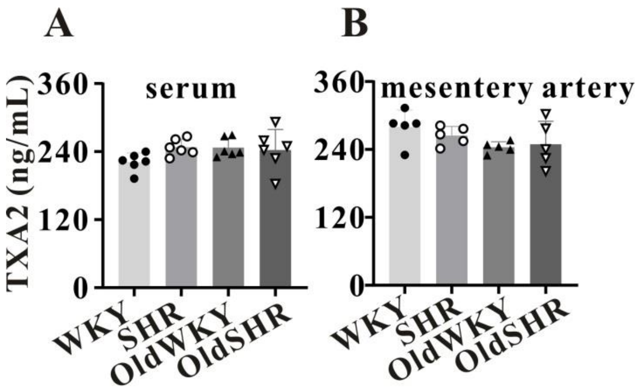


**Supplemental Figure 3. Determination of TXA2 level in serum and mesentery artery by ELISA.**

No significant changes were detected in serum or vascular tissue. Samples were collected from 5 to 6 rats in each group.

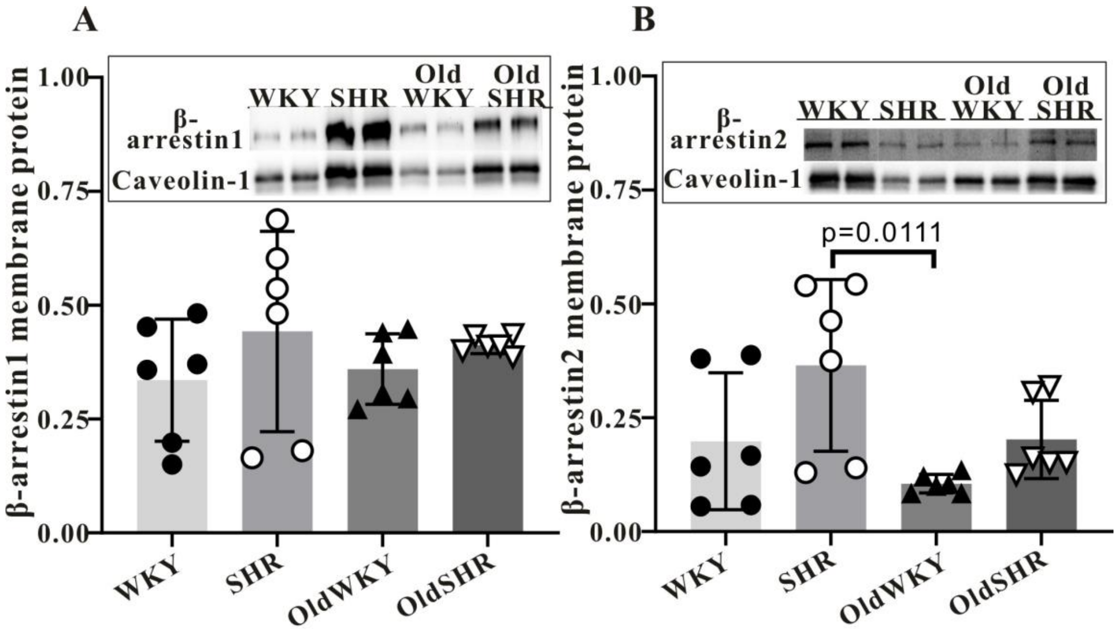


**Supplemental Figure 4. Expression of β-arrestin 1 (A) and β-arrestin 2 (B) in the mesentery artery of WKY, SHR, OldWKY, and OldSHR.** Intact mesentery arteries of the WKY, SHR, OldWKY, and OldSHR were obtained from 6 rats in each group.
